# Acute Aerobic Exercise Ameliorates Cravings and Inhibitory Control in Heroin Addicts: Evidence From Event-Related Potentials and Frequency Bands

**DOI:** 10.3389/fpsyg.2020.561590

**Published:** 2020-09-29

**Authors:** Dongshi Wang, Ting Zhu, Jiachen Chen, Yingzhi Lu, Chenglin Zhou, Yu-Kai Chang

**Affiliations:** ^1^Faculty of Sports Science, Ningbo University, Ningbo, China; ^2^Center for Mental Health and Education, Ningbo City College of Vocational Technology, Ningbo, China; ^3^School of Psychology, Shanghai University of Sport, Shanghai, China; ^4^Department of Physical Education, National Taiwan Normal University, Taipei, Taiwan; ^5^Institute for Research Excellence in Learning Science, National Taiwan Normal University, Taipei, Taiwan

**Keywords:** acute aerobic exercise, heroin addicts, inhibitory control, N2d, theta

## Abstract

**Objective:**

Aerobic exercise is considered a potential adjunctive treatment for heroin addicts, but little is known about its mechanisms. Less severe cravings and greater inhibitory control have been associated with reduced substance use. The aim of the current study was to determine the effects, as measured by behavioral and neuroelectric measurements, of acute aerobic exercise on heroin cravings and inhibitory control induced by heroin-related conditions among heroin addicts.

**Design:**

The present study used a randomized controlled design.

**Methods:**

Sixty male heroin addicts who met the DSM-V criteria were recruited from the Isolated Detoxification Center in China and randomly assigned to one of two groups; one group completed a 20-min bout of acute stationary cycle exercise with vigorous intensity (70–80% of maximum heart rate, exercise group), and the other group rested (control group). The self-reported heroin craving levels and inhibitory control outcomes (measured by a heroin-related Go/No-Go task) were assessed pre- and post-exercise.

**Results:**

The heroin craving levels in the exercise group were significantly attenuated during, immediately following, and 40 min after vigorous exercise compared with before exercise; moreover, during exercise, a smaller craving was observed in the exercise group than in the control group. Acute exercise also facilitated inhibition performance in the No-Go task. After exercise, the participants’ accuracy, the N2d amplitudes, and the theta two band spectral power during the No-Go conditions were higher in the exercise group than in the control group. Interestingly, significant correlations between the changes in these sensitive measurements and the changes in cravings were observed.

**Conclusions:**

This is the first empirical study to demonstrate that aerobic exercise may be efficacious for reducing heroin cravings and promoting inhibitory control among heroin addicts.

## Introduction

Heroin addiction is becoming more common globally and is thus a major public health problem. The relapse rate of heroin addicts, which is considered a major persistent obstacle to rehabilitation treatment (Stewart, 2008), remains high after 1 year of treatment programs (Carroll et al., 1994), and new approaches to treatment are needed. Previous research suggests that aerobic exercise may be a useful adjunctive treatment for drug addicts (including heroin addicts), although the evidence is from exploratory research (Smith and Lynch, 2012; Pareja-Galeano et al., 2013). In creating an evidence base, it is important to establish plausible mechanisms. Previous studies suggest that more severe cravings for substances and lower levels of inhibitory control predict substance use; therefore, treatments that reduce cravings and promote inhibitory control may be useful (Volkow et al., 2016). The beneficial effects of aerobic exercise on cravings for other substances [e.g., methamphetamine (Wang et al., 2015), cigarettes (De La Garza et al., 2016; Abrantes et al., 2017), and alcohol (Hallgren et al., 2017)] have been widely reported. Only a few empirical studies have reported that heroin cravings become less severe after acute aerobic exercise (Bailey et al., 2011). Therefore, it is important to understand the effects of aerobic exercise on heroin addiction and related psychological mechanisms.

Although multiple factors contribute to the probability of a craving, evidence suggests that executive function impairment contributes to heroin use and relapse from treatment (Morie et al., 2014; Stevens et al., 2014). Chronic heroin abuse is associated with executive dysfunction, particularly inhibitory control dysfunction (Stevens et al., 2014; Volkow et al., 2016). Inhibitory control impairment makes it difficult for individuals to resist the temptation of drug cues and impulsive thoughts and behaviors of intake. Moreover, neuroimaging studies have further demonstrated that, relative to healthy groups, heroin addict groups exhibit abnormal structures and lower levels of activation in brain regions related to inhibitory control during the Go/No-Go task, including impairments in the bilateral medial prefrontal gyrus, bilateral anterior cingulate cortex (ACC), dorsolateral prefrontal cortex (DPFC), left middle frontal gyrus (MFG), and left insular and bilateral inferior frontal gyrus (IFG) (Fu et al., 2008; Luijten et al., 2014; Moningka et al., 2018). Inhibitory control failure and associated brain dysfunction are key underlying factors of relapse and addiction (Su et al., 2017). Therefore, inhibitory control may be an important factor related to aerobic exercise in reducing the risk of relapse in heroin addicts.

A substantial body of systematic studies on acute aerobic exercise and executive function interactions have shown that acute aerobic exercise can positively modulate inhibitory control (Chang et al., 2012; Basso and Suzuki, 2017; Li et al., 2017). Moreover, many empirical studies have also shown that acute aerobic exercise has a positive effect on inhibitory control in various paradigms, including the Go/No-Go task (Kamijo et al., 2004), stop-signal task (Chu et al., 2015), and flanker task (Hillman et al., 2006). This beneficial effect of acute exercise has also been observed in other groups of individuals with brain dysfunction, such as those with attention-deficit hyperactivity disorder (Gapin et al., 2015; Mehren et al., 2019), Alzheimer’s and Parkinson’s disease (Paillard et al., 2015), and aging (Barnes, 2015). In addition, neuroimaging studies have revealed significant levels of activation in some brain regions associated with inhibitory control after acute aerobic exercise, including the ACC, IFG, and vermis cerebelli (Krafft et al., 2013; Fontes et al., 2015). More importantly, acute aerobic exercise facilitates inhibitory control in individuals with methamphetamine addiction (Wang et al., 2015), and this previous study demonstrated improved inhibitory control after acute aerobic exercise, providing preliminary evidence that acute aerobic exercise is beneficial for individuals with drug addictions. From the above results, functional complementarity and majority overlapping in the cortical structure may exist, indicating that it is highly likely that acute aerobic exercise can modulate inhibitory control in heroin addicts.

The vast majority of electroencephalogram (EEG) studies have characterized inhibitory control dysfunction in heroin addicts using event-related potentials (ERPs) associated with the Go/No-Go task (Morie et al., 2014; Steele et al., 2014; Yang et al., 2015; Su et al., 2017). These studies have shown that the N2 component is sensitive in measuring the dysfunction of heroin addicts under No-Go conditions. The largest negative-going peak over the fronto-central cortex occurs within 200–300 ms of stimulus onset in the N2 component. The N2 can reflect features of conflict monitoring; features of cognitive control processing (Falkenstein, 2006; Folstein and Van Petten, 2008; Groom and Cragg, 2015), which is a top-down cognitive mechanism in the early stages of inhibitory control; and a reduction in the N2 amplitude during the No-Go task, which is a sensitive indicator of impaired inhibition in heroin addicts (Morie et al., 2014; Su et al., 2017). Moreover, to more clearly highlight the effects on inhibitory control processing that were mentioned above, N2d, which is defined as the difference in signals from the No-Go trials and those from the Go trials, is widely used (Cao et al., 2017; Su et al., 2017). In addition to the ERP components mentioned above that are related to inhibitory control, the changes in spectral power that occur with performance in the Go/No-Go task have also been studied by many researchers. The changes in the spectral power of EEG signals reflect the processing of neuron rhythm adjustments induced by a stimulus, such as an excitatory or inhibitory stimulus, and the spectral power in different frequency bands are often related to various cognitive activities performed by human beings (Klimesch et al., 2007; Nguyen et al., 2017). Moreover, the theta (4–8 Hz) bands are strongly affected by the Go/No-Go paradigm (Cavanagh and Frank, 2014; Nguyen et al., 2017). The spectral power of the event-related theta band (4–8 Hz) is related to fronto-limbic interactions (Karakaş et al., 2000) and is associated with high-order cognitive processes involved in top-down control (Cavanagh et al., 2012; Cavanagh and Frank, 2014) and response conflict (Cohen and Cavanagh, 2011). Previous studies have suggested that lower levels of theta-band power are associated with poorer inhibitory functions (Motlagh et al., 2017; Fielenbach et al., 2019).

In the current study, we used a randomized controlled design to test the effects of acute aerobic exercise on cravings and inhibitory control among heroin addicts. The intensity of aerobic exercise used was vigorous intensity (70–80% of maximum heart rate), which is the most suitable model for heroin addicts (Bailey et al., 2011; Colledge et al., 2017). Additionally, we evaluated inhibitory control in heroin addicts from behavioral and neuroelectric perspectives (e.g., ERPs and spectral power) with the Go/No-Go task, which is related to heroin cues. We hypothesized that acute aerobic exercise with vigorous intensity would decrease heroin cravings, improve behavioral performance, and facilitate neuroelectric activation-related inhibitory control.

## Materials and Methods

### Participants

Sixty male heroin addicts aged between 20 and 40 years were recruited from the Yunnan Compulsory Isolated Detoxification Center in China. All participants met the criteria for Diagnosis and Statistics of Mental Disorder 5th edition (DSM-V) for heroin addicts. The inclusion criteria included (a) intact color vision, (b) a normal level of intelligence, (c) the absence of a history of neurological diseases or physical disabilities, and (d) the absence of medical contraindications for high-intensity exercise, as determined by the Physical Activity Readiness Questionnaire (PAR-Q) (American College of Sports Medicine, 2013). The participants were randomly assigned to either the aerobic exercise or sedentary control group. The demographic characteristics of the two groups are summarized in Table 1. Before participating in the study, all participants signed a consent form, which was approved by the Ethics Committee of Ningbo University.

**TABLE 1:** Participants’ demographic characteristic (mean ± SD/*n*+%).

	**Exercise (*n* = 30)**	**Control (*n* = 30)**	***t/*χ^2^**
Demographic			
Age (years)	32.73 ± 7.15	32.40 ± 7.76	0.86
Height (cm)	170.48 ± 7.03	168.44 ± 5.62	0.23
Weight (kg)	61.88 ± 8.81	62.42 ± 9.11	0.42
IQ (PR)	10.50 ± 10.03	10.50 ± 8.34	1.00
SES			
Marriage			
Single	10 (33.33%)	10 (29.4%)	0.84
Married	11 (36.67%)	12 (35.3%)	
Live together	1 (3.33%)	4 (11.8%)	
Divorce	6 (20.00%)	7 (20.6%)	
Widowed	2 (6.67%)	1 (2.9%)	
Education			
Elementary	16 (53.33%)	13 (43.33%)	0.67
Junior	13 (43.33%)	15 (50.00%)	
Senior	1 (3.33%)	2 (6.67%)	
Occupation			
Farm laborer	16 (53.33%)	13 (43.33%)	0.22
Self-employed	3 (10.00%)	10 (33.33%)	
Manual worker	2 (6.67%)	1 (3.33%)	
General staff	1 (3.33%)	2 (6.67%)	
Civil servants	1 (3.33%)	0 (0%)	
Peasant laborer	4 (13.33%)	3 (3.33%)	
Not employed	3 (10%)	1 (3.33%)	
Family income (100 yuan/month)	38.13 ± 31.84	28.88 ± 28.65	0.24
Smoke data			
Usage (*n*)	30 (100%)	30 (100%)	1.00
Duration (years)	16.46 ± 6.73	17.68 ± 7.72	0.48
Frequency (cigarette/day)	20.27 ± 13.10	21.93 ± 13.82	0.63
Alcohol data			
Usage (*n*)	18 (60%)	18 (60%)	1.00
Duration (year)	11.60 ± 8.24	11.64 ± 9.84	0.98
Frequency (time/week)	2.80 ± 4.05	2.10 ± 3.19	0.46
Heroin data			
Usage (g/time)	0.35 ± 0.40	0.35 ± 0.31	0.97
Frequency (time/month)	66.60 ± 42.19	79.07 ± 61.45	0.37
Duration (year)	7.00 ± 6.07	7.83 ± 5.68	0.59
Relapse (time)	3.27 ± 3.56	3.93 ± 9.11	0.71
Fitness and physical activity			
DBP (mmHg)	73.26 ± 9.10	69.05 ± 13.27	0.12
SBP (mmHg)	127.69 ± 15.11	125.65 ± 14.21	0.52
Resting HR (bpm)	76.12 ± 11.36	75.24 ± 11.32	0.43
VO_2max_ (ml/kg/min)	2.47 ± 1.17	2.59 ± 1.00	0.71
BMI (kg/m^2^)	21.29 ± 1.74	22.01 ± 3.17	0.56
IPAQ (1000 MET/week)	20.68 ± 16.81	19.37 ± 17.59	0.78
Emotional status			
SAS	53.43 ± 9.95	52.13 ± 8.78	0.61
SDS	50.53 ± 14.80	47.70 ± 12.31	0.42

*SES, socioeconomic status; resting HR, resting heart rate; DBP, diastolic blood pressure; SBP, systolic blood pressure; IQ, reven’s standard test of intelligence (percentile rank, PR); IPAQ, international physical activity questionnaire (100 MET/week); SAS, self-rating anxiety scale; SDS, self-rating depression scale. VO_2max_ was evaluated using an Astrand-Rhyming cycle ergometer-based cardiorespiratory fitness test (evaluated based on VO_2max_).*

### Measurements

#### Cravings

The self-reported craving level measured the strength of heroin desire using a visual analogue scale (VAS) (Sayette et al., 2000). The VAS rated on a scale from 0 = “no craving at all” to 10 = “extremely craving”. After watching a series of randomly presented heroin-related images including forms of heroin, heroin paraphernalia, and heroin-related scenes on iPad, the participant was required to mark on a 10-cm line.

#### Inhibitory Control

**FIGURE 1:**
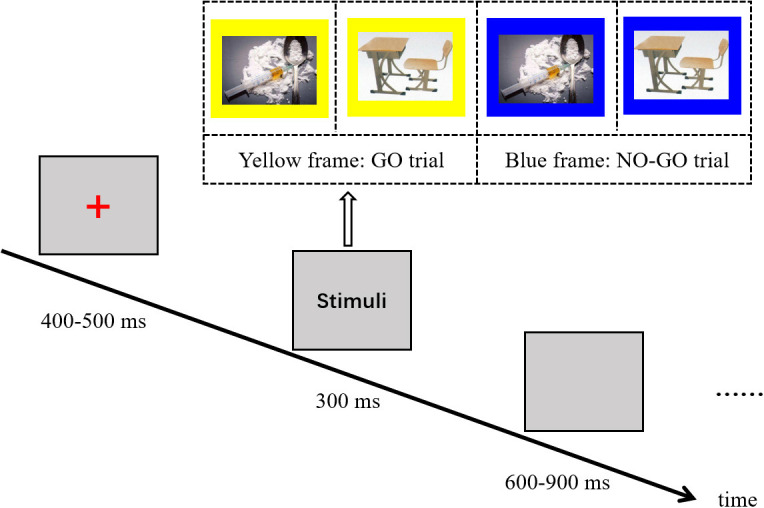
Illustration of the heroin-related Go/No-Go task. Press the space bar as quickly as possible when the stimulus interface presents an image with a yellow frame (i.e., Go trial: Go-Neutral or Go-Heroin) but withhold the response when an image with a blue frame is presented (i.e., No-Go trial: No-Go-Neutral or No-Go-Heroin).

For the Go/No-Go task, heroin cue images and neutral cue images with either blue or yellow frames were presented at a visual angle of 10.3° × 14.8° via E-prime software (E-prime 2.0, Psychology Software Tools, Inc., United States), as described in our previous work, to assess the inhibitory control (Wang et al., 2015). Fifteen neural images (valence: 5.23; arousal: 5.33) were selected from the Chinese Affective Picture System (Lu et al., 2005). The fifteen heroin-related images (valence: 4.12; arousal: 4.37) depicted various forms of heroin, heroin paraphernalia, and heroin-related scenes, which were selected based on the results of a nine-point self-rating scale from another 36 heroin addicts. The heroin-related and neutral images were carefully selected so that they matched in terms of luminance, contrast, and spatial frequency. Each image with a frame was rapidly presented one by one on a gray background with a presentation time of 300 ms and an interstimulus interval that randomly varied from 600 to 900 ms (see Figure 1). The participants were instructed to respond to images with a yellow frame (i.e., Go trial: Go-Neutral or Go-Heroin) as quickly as possible by pressing the space bar but to withhold their response when an image had a blue frame (i.e., No-Go trial: No-Go-Neutral or No-Go-Heroin). Three blocks were administered, consisting of 150 Go trials and 50 No-Go trials with equal probability of occurring in a quasi-random order.

### Procedure

Several types of background information were gathered during the baseline tests, including the participants’ demographic characteristics, socioeconomic statuses, medical histories, smoking habits, alcohol consumption habits, heroin dependence, and physical activity; their emotional statuses were assessed via the Self-rating Depression Scale (SDS) and the Self-rating Anxiety Scale (SAS). The eligible participants then underwent the Astrand-Rhyming fitness test, and their body mass index (BMI) values were recorded.

The heroin addicts were randomly assigned to the exercise group or control group with the aid of a computer algorithm^1^ after baseline testing. The participants in the exercise group were engaged in a vigorous-intensity aerobic exercise program that involved a 5-min warm-up, a 20-min main exercise period, and a 5-min cool down. During the main exercise period, the participants were instructed to complete a bout of stationary cycle exercise and adjust the load so that their the heart rate (HR) remained within 70–80% of their maximum HR (HR max = 206.9–0.67 × age). HR was monitored using PolarRCX3 (Polar Company, Finland). The participants’ HR and rating of perceived exertion (RPE) were recorded every 2 min throughout the exercise period. The mean HR for this exercise was 146.36 ± 4.39 bpm, which equated to 78.61 ± 1.57% of the maximum HR. Additionally, the mean RPE score was 14.93 ± 1.08. Participants in the control group were required to read about heroin treatments in a quiet room for 30 min.

The VAS score was measured at several time points, including before the cognitive pre-test, before the warm-up, during the exercise period, immediately after the exercise period, and after the cognitive post-test. In addition, before the warm-up and after the exercise period, when their HR returned to within 110% of their resting HR levels, the participants were required to complete the heroin-related Go/No-Go task with an EEG cap. The total duration of the experimental procedure was approximately 100 min.

### Electroencephalographic Recording and Data Processing

The EEG signals were recorded using a 32-channel Ag/AgCl electrode cap (10–20 International System), a Brain Amp amplifier, and a Brain Vision Recorder 2.0 system (Brain Products Company, Germany). The EEG signals were referenced to FCz and grounded to the AFz electrode. Horizontal and vertical electrooculography (HEOG and VEOG) signals were recorded from the outer canthi of the right eye and the infraorbital region of the left eye, respectively. In addition, the EEG signals were amplified using a bandpass filter from 0.01 to 100 Hz and sampled at a rate of 1000 Hz. The impedance of each electrode remained below 5 kΩ.

We used the Brain Vision Analyzer 2.0 system (Brain Products Company, Germany) to analyze the EEG data offline. After the EEG signals were re-referenced to the average signal from the bi-mastoid electrodes, they were bandpass filtered from 0.5 to 100 Hz, and EOG artifacts were corrected by the independent component analysis (ICA) algorithm. Trials with artifacts that exceeded ± 100 μv were rejected. The EEG was segmented into the epoch using stimulus locked from *−*200 to 800 ms for correct trials, and baseline correction was performed using the 200-ms pre-stimulus period. The N2 and difference in N2 waves (i.e., N2d) were measured. In addition, the EEG signals were analyzed by fast Fourier transform before overlay averaging, and then, the spectrum power of the frequency bands for theta 1 (4–6 Hz) and theta 2 (6–8 Hz) was obtained.

### Statistical Analysis

Independent *t*-test and Chi-square test were employed to analyze differences in demographic characteristics between the exercise and control groups. Regarding cravings, 2 (group: exercise and control) × 5 (time point: pre-test, pre-exercise, during exercise, post-exercise, and follow-up) repeated-measures analysis of variance (RM-ANOVA) was performed. Regarding the behavioral data (including Go-RT, Go accuracy, and No-Go accuracy) for the heroin-related Go/No-Go task, 2 (group) × 2 (time point: pre-test and post-test) × 2 (condition: heroin cue and neutral cue) RM-ANOVA was performed. Regarding the N2 data and spectrum power data, 2 (group) × 2 (time point) × 4 (condition: heroin Go, neutral Go, heroin No-Go, and neutral No-Go) × 4 (site: Fz, FCz, Cz, and Pz) RM-ANOVA was performed, and the 2 (group) × 2 (time point) × 4 (site) RM-ANOVA was performed to analyze the N2d data from the two conditions. Additionally, the correlations between the changes in the main outcomes, defined as the differences between the post-test and pre-test (represented by the symbol Δ), were analyzed using two-tailed Pearson correlation analysis. Greenhouse-Geisser correction and *post hoc* comparisons using *t*-tests with Bonferroni correction were used. Partial eta-squared (η_p_^2^) values were reported for results with significant effect sizes, and the statistical significance level was set to be 0.05.

## Results

### Craving Measures

**FIGURE 2:**
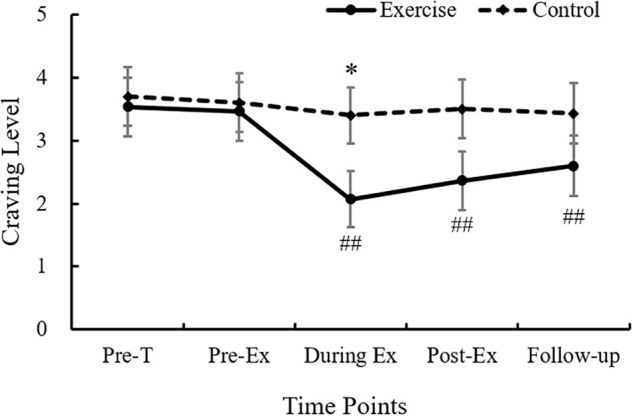
Craving level alterations before test, before exercise, during exercise, immediately after exercise, and 60 min after exercise in two groups. ^∗^Represents a significant difference between exercise and control group, *p* < 0.05. ##represents craving at this time point is less than that at pre-task and pre-exercise in exercise group, *p* < 0.01.

The analysis of craving revealed a significant main effect of time point [*F*(4,232) = 18.74, *p* < 0.001, η*_*p*_*^2^ = 0.24], and an interaction effect of group × time point [*F*(4,232) = 9.42, *p* < 0.01, η*_*p*_*^2^ = 0.14]. Follow-up comparison showed that the craving of exercise group decreased from during exercise to following exercise (*p*s < 0.01). Additionally, less craving score in the exercise group (2.06 ± 1.76) relative to control group (3.40 ± 2.96) was observed during exercise time point (*p* = 0.04 < 0.05, see Figure 2).

### Behavioral Data

In the heroin-related Go/No-Go task, regarding Go-RT, only the main effects of the time point [*F*(1,58) = 76.39, *p* < 0.001, η*_*p*_*^2^ = 0.57] and condition [*F*(1,58) = 4.20, *p* = 0.045 < 0.05, η*_*p*_*^2^ = 0.07] were revealed. No other effects were observed (see Table 2).

**TABLE 2:** Behavioral, neuroelectric, and spectral power data at channel FCz for the heroin-related Go/No-Go task during the treatment session (mean ± SD).

**Variable**	**Exercise**		**Control**	
	
	**Pre-test**	**Post-test**	**Pre-test**	**Post-test**
Heroin-Go RT (ms)	372.59 ± 50.96	348.79 ± 46.42	359.14 ± 45.41	342.36 ± 44.23
Neutral-Go RT (ms)	370.99 ± 50.32	348.51 ± 45.41	357.26 ± 45.96	341.01 ± 41.83
Heroin-Go accuracy (%)	95.90 ± 8.08	97.07 ± 5.99	98.40 ± 1.96	97.60 ± 3.61
Neutral-Go accuracy (%)	95.77 ± 8.42	96.47 ± 6.39	98.20 ± 2.25	98.13 ± 2.81
Heroin-No-Go accuracy (%)	86.07 ± 8.47	91.57 ± 6.69	89.41 ± 6.44	89.37 ± 7.05
Neutral-No-Go accuracy (%)	88.20 ± 6.37	90.87 ± 6.44	89.30 ± 6.58	90.00 ± 6.75
Amplitude (μV)				
Heroin-Go-N2	−4.00 ± 3.41	−3.40 ± 3.87	−4.18 ± 3.40	−3.72 ± 3.22
Heroin-No-Go-N2	−6.92 ± 4.41	−8.08 ± 5.33	−7.08 ± 3.38	−6.67 ± 3.77
Heroin-N2d	−4.61 ± 3.04	−6.78 ± 3.90	−4.52 ± 2.79	−4.53 ± 3.66
Neutral-Go-N2	−3.72 ± 3.62	−3.02 ± 4.10	−3.76 ± 3.60	−3.31 ± 2.92
Neutral-No-Go-N2	−6.48 ± 4.55	−8.04 ± 5.37	−6.25 ± 3.61	−6.72 ± 3.74
Neutral-N2d	−4.33 ± 2.59	−7.05 ± 3.39	−4.28 ± 3.17	−5.16 ± 3.67
Latency (ms)				
Heroin-Go-N2	249.63 ± 43.12	246.27 ± 46.06	264.70 ± 60.21	264.70 ± 55.86
Heroin-No-Go-N2	254.13 ± 28.03	249.20 ± 21.67	253.57 ± 25.21	251.70 ± 28.57
Heroin-N2d	259.06 ± 29.85	257.80 ± 23.53	262.23 ± 28.82	267.03 ± 40.58
Neutral-Go-N2	251.77 ± 46.63	253.80 ± 51.61	271.70 ± 67.17	259.37 ± 53.16
Neutral-No-Go-N2	255.23 ± 27.50	248.67 ± 20.53	255.27 ± 28.24	250.70 ± 27.03
Neutral-N2d	264.70 ± 36.03	264.27 ± 32.79	266.37 ± 36.50	257.26 ± 26.14
Theta 1 power (4–6 Hz; μV^2^)				
Heroin-Go	2.08 ± 1.37	2.41 ± 1.58	2.04 ± 1.61	2.34 ± 1.80
Heroin-No-Go	4.37 ± 2.72	4.95 ± 2.69	3.66 ± 2.20	4.53 ± 2.51
Neutral-Go	2.08 ± 1.34	2.48 ± 1.62	2.01 ± 1.37	2.14 ± 1.79
Neutral-No-Go	4.24 ± 2.63	4.86 ± 3.14	4.01 ± 2.23	4.23 ± 2.81
Theta 2 power (6–8 Hz; μV^2^)				
Heroin-Go	1.330.83	1.641.31	1.200.90	1.190.73
Heroin-No-Go	2.00 ± 1.18	2.68 ± 1.54	1.97 ± 1.14	1.80 ± 0.99
Neutral-Go	1.15 ± 0.70	1.41 ± 1.14	1.07 ± 0.51	1.26 ± 0.73
Neutral-No-Go	1.96 ± 0.96	2.59 ± 1.71	1.85 ± 1.21	2.07 ± 0.89

Regarding Go accuracy, neither main effects nor interaction effects were observed (see Table 2).

**FIGURE 3:**
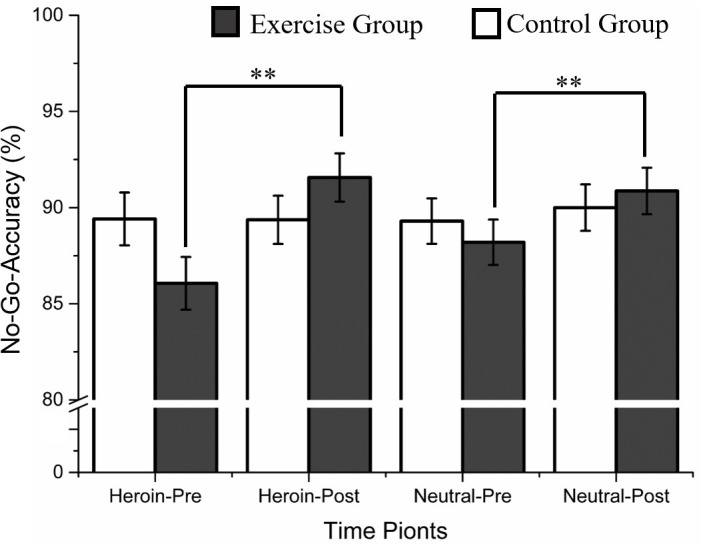
Accuracy of heroin-related Go/No-Go task as a function of group and time point. ^∗∗^Represents a significant difference between pre- and post-test in heroin cue No-Go and neutral cue No-Go condition, *p* < 0.01.

Regarding No-Go accuracy, a main effect of time point [*F*(1,58) = 14.99, *p* < 0.001, η*_*p*_*^2^ = 0.21] and a marginal three-way interaction effect of time point × condition × group [*F*(1,58) = 3.54, *p* = 0.06, η*_*p*_*^2^ = 0.06] were observed. Follow-up analysis indicated that greater accuracies in exercise group during the heroin cue No-Go and neutral cue No-Go conditions in post-test compared with pre-test were observed (*p*s < 0.001, see Table 2 and Figure 3).

### ERP Data

Regarding N2 latency, only a main effect of site [*F*(3,174) = 5.09, *p* < 0.01, η*_*p*_*^2^ = 0.08], with an earlier latency for the *Cz* site than for the *Pz* site, was observed (*p* < 0.05). Since no differences in N2 latency among groups or conditions were observed, the following statistical analysis of the ERP data is focused on the amplitude.

**FIGURE 4:**
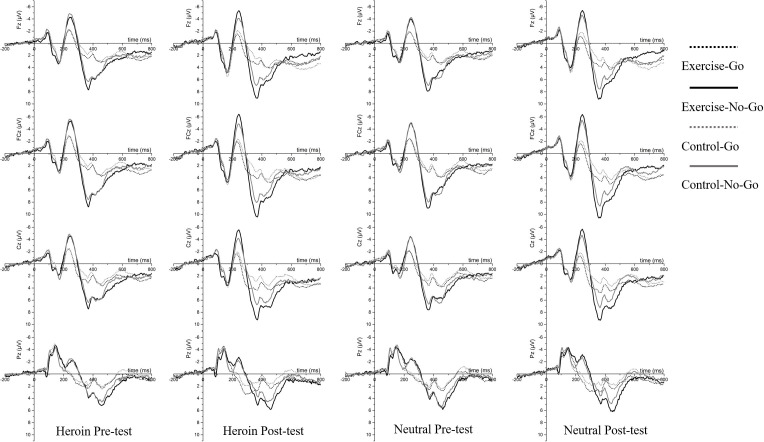
Grand average ERPs between exercise group (Black) and control group (Gray) at Fz, FCz, Cz, and Pz for Go trials (Dot lines) and No-Go trials (Solid lines) for four tests: Heroin cue pre- and post-test and Neutral cue pre- and post-test.

Regarding N2 amplitude, the main effects of site [*F*(3,174) = 10.37, *p* < 0.001, η*_*p*_*^2^ = 0.15] and condition [*F*(3,174) = 90.39, *p* < 0.001, η*_*p*_*^2^ = 0.61] were observed; importantly, the interaction effects of time point × condition × site [*F*(9,522) = 4.96, *p* < 0.01, η*_*p*_*^2^ = 0.08] and time point × condition × group [*F*(3,174) = 3.17, *p* = 0.04 < 0.05, η*_*p*_*^2^ = 0.05] were observed. Follow-up analysis revealed that the N2 amplitudes for Fz, FCz, and Cz in the neutral No-Go condition were larger during the post-test than during the pre-test (*p*s < 0.05). Moreover, the N2 amplitudes in the exercise group in the heroin No-Go and neutral No-Go conditions were larger during the post-test than during the pre-test (*p*s < 0.05). Furthermore, the N2 amplitudes were generally larger for the No-Go conditions than for the Go conditions (*p*s < 0.01, see Table 2 and Figure 4).

**FIGURE 5:**
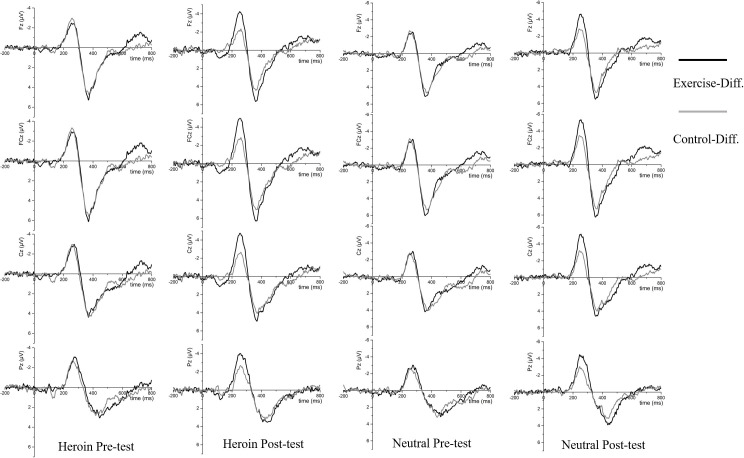
The difference waveforms between No-Go and Go ERPs in exercise and control groups for four tests: Heroin cue pre- and post-test and Neutral cue pre- and post-test, respectively.

Regarding heroin cue N2d amplitude, the main effects of time [*F*(1,58) = 12.72, *p* < 0.01, η*_*p*_*^2^ = 0.18] and site [*F*(3,174) = 3.94, *p* < 0.05, η*_*p*_*^2^ = 0.06] were observed; importantly, the interaction effect of time point × site × group [*F*(3,174) = 3.43, *p* < 0.05, η*_*p*_*^2^ = 0.06] was observed. Follow-up analysis revealed that the N2d amplitudes at Fz, FCz, and Cz in the exercise group were larger during the post-test than during the pre-test (*p*s < 0.001); moreover, the N2d amplitudes at Fz, FCz, and Cz during the post-test were larger in the exercise group than in the control group (*p*s < 0.05, see Table 2 and Figure 5).

Regarding neutral cue N2d amplitude, the main effects of time [*F*(1,58) = 28.09, *p* < 0.001, η*_*p*_*^2^ = 0.33] and site [*F*(3,174) = 4.76, *p* < 0.01, η*_*p*_*^2^ = 0.08] were observed; importantly, the interaction effect of time point × site × group [*F*(3,174) = 5.24, *p* < 0.05, η*_*p*_*^2^ = 0.08] was observed. Follow-up analysis revealed that the N2d amplitudes at Fz, FCz, and Cz in the exercise group were larger during the post-test than during the pre-test (*p*s < 0.01); moreover, the N2d amplitudes for FCz and Cz (*p*s < 0.05) and N2d amplitude at Fz (*p* = 0.06) during the post-test were larger and marginally significantly larger, respectively, in the exercise group than in the control group (see Table 2 and Figure 5).

### Spectral Power Data

Regarding theta 1 power, the main effects of time [*F*(1,58) = 4.38, *p* < 0.05, η*_*p*_*^2^ = 0.07], site [*F*(3,174) = 22.19, *p* < 0.001, η*_*p*_*^2^ = 0.28], and condition [*F*(3,174) = 63.64, *p* < 0.001, η*_*p*_*^2^ = 0.52] were observed; importantly, the interaction effect of time point × site × condition [*F*(9,522) = 2.74, *p* < 0.05, η*_*p*_*^2^ = 0.05] was observed. Follow-up analysis revealed that the theta 1 power outputs at Fz, FCz, and Cz in the heroin No-Go condition were larger during the post-test than during the pre-test (*p*s < 0.05), and the theta 1 power outputs at Fz, FCz, and Cz were larger in the No-Go condition than in the Go condition (*p*s < 0.001).

**FIGURE 6:**
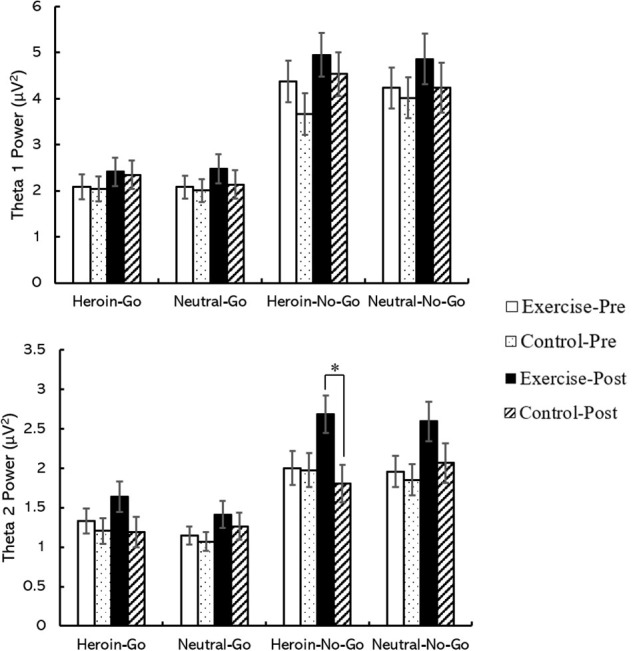
The spectral powers of FCz site between the exercise group and the control group in the heroin-related Go/No-Go task. ^∗^Represents a significant difference between exercise group and control group in post-test in heroin cue No-Go condition, *p* < 0.05.

Regarding theta 2 power, the main effects of time [*F*(1,58) = 9.89, *p* < 0.001, η*_*p*_*^2^ = 0.15], site [*F*(3,174) = 33.45, *p* < 0.001, η*_*p*_*^2^ = 0.37], and condition [*F*(3,174) = 39.31, *p* < 0.001, η*_*p*_*^2^ = 0.40] were observed; importantly, a marginally significant interaction effect of time point × site × condition × group [*F*(9,522) = 1.75, *p* = 0.07, η*_*p*_*^2^ = 0.03] was observed. Follow-up analysis revealed that the theta 2 power outputs for Fz, FCz, and Cz in the heroin No-Go condition during the post-test were larger in the exercise group than in the control group (*p*s < 0.05); in addition, the theta 2 power outputs for Fz, FCz, and Cz for all groups and times were larger during the No-Go condition than during the Go condition (*p*s < 0.05). Furthermore, the theta 2 power outputs for Fz, FCz, and Cz for all conditions in the exercise group were larger during the post-test than during the pre-test (*p*s < 0.05, see Table 2 and Figure 6).

### Correlational Analyses

**FIGURE 7:**
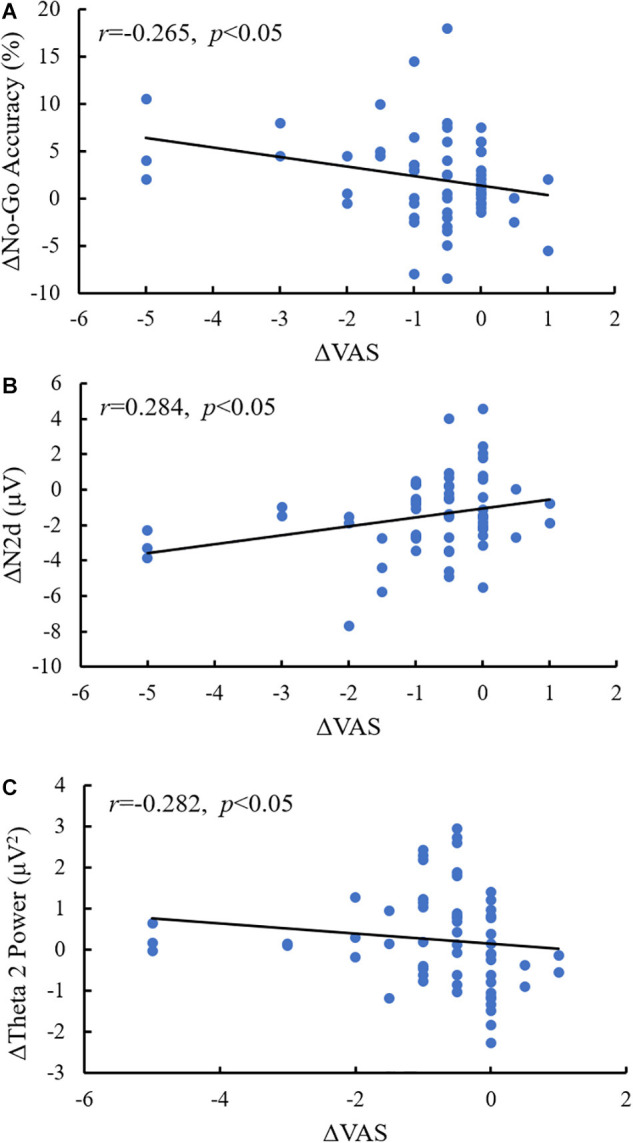
Scatter plots for the correlation. **(A)** Correlation between ΔNo-Go accuracy and ΔVAS; **(B)** correlation between ΔN2d amplitude and ΔVAS; and **(C)** correlation between Δtheta 2 power and ΔVAS.

The magnitude of ΔVAS was significantly correlated with ΔN2d amplitude (*r* = 0.284, *p* = 0.03 < 0.05), but it was negatively correlated with ΔNo-Go accuracy (*r* = *−*0.265, *p* = 0.04 < 0.05) and Δtheta 2 power (*r* = *−*0.282, *p* = 0.03 < 0.05) (see Figure 7).

## Discussion

### Acute Aerobic Exercise Reduces Heroin Cravings

Our study provides strong evidence that acute aerobic exercise with vigorous intensity affects heroin cravings and heroin-related inhibitory control, as determined by behavioral and neuroelectric measurements. We found that heroin cravings began to significantly decrease during aerobic exercise, and this effect persisted for 40 min after exercise. These findings are similar to those in a prior study that observed reduced cravings for opiates in patients undergoing methadone maintenance treatment after 20 min of acute aerobic exercise with vigorous intensity (Bailey et al., 2011). Moreover, a similar effect of acute aerobic exercise with vigorous intensity on cravings has been observed in other types of addicts [i.e., methamphetamine addicts (Wang et al., 2016) and smokers (Everson et al., 2008; Scerbo et al., 2010)]. In summary, acute aerobic exercise with vigorous intensity can be used to treat heroin addicts and other types of addicts to ameliorate cravings.

There are different opinions about the underlying mechanisms by which aerobic exercise reduces the cravings of heroin addicts. Many researchers believe that these beneficial effects may be attributable to physiological and neurobiological mechanisms, in which aerobic exercise activates and modulates the neurotrophic factors (i.e., brain-derived neurotrophic factor) associated with addiction (Pareja-Galeano et al., 2013). However, this gene transcription may not occur synchronously in an acute aerobic exercise intervention (Tsai et al., 2014; Chang et al., 2017). Another plausible explanation is that acute aerobic exercise alters heroin addicts’ emotions and induces stress to improve performance, which reduces cravings (Everson et al., 2008; Scerbo et al., 2010). However, individual emotions and stress are actually regulated by advanced cognitive function (i.e., executive function) (Heatherton and Wagner, 2011; Volkow et al., 2016). Combining these explanations of psychological mechanisms and the current theory of executive dysfunction in heroin addicts, we deduce that cognitive function (i.e., inhibitory control) may play an important role in the process of reducing heroin cravings.

### Acute Aerobic Exercise and Inhibitory Control: Behavioral Measures

The behavioral data demonstrate that participants’ accuracy during the Go/No-Go task with heroin and neutral cues improves in the No-Go condition, but not in the Go condition, following acute aerobic exercise. The findings are similar to those in a prior study showing that acute aerobic exercise improves the No-Go accuracy of methamphetamine addicts (Wang et al., 2015, 2016). Additionally, no significant differences were observed between heroin cues and neutral cues in terms of the No-Go task accuracy in the post-test. This finding suggests that acute aerobic exercise indiscriminately promotes inhibitory control in both general situations and heroin cue situations. Beneficial effects of acute aerobic exercise for inhibitory control in cognitive dysfunction individuals have been reported in prior studies (Chang et al., 2014; Chuang et al., 2015). This evidence also confirms that acute aerobic exercise has a positive effect on inhibitory control in heroin addicts. Interestingly, the current study demonstrates that ΔVAS is negatively correlated with ΔNo-Go accuracy (*r* = *−*0.265). This result preliminarily suggests that acute aerobic exercise is strongly related with improvement in inhibitory control and reductions in heroin cravings in heroin addicts. Because inhibitory control dysfunction is an important predictor of successful detoxification (Parvaz et al., 2011; Garavan and Weierstall, 2012), these behavioral measurement findings are very valuable.

### Acute Aerobic Exercise and Inhibitory Control: ERP Measures

The N2 amplitudes were larger in the No-Go conditions than in the Go conditions in both groups and at both time points, indicating that the conflict monitoring system was activated and that our experimental procedure was successful. Interestingly, the N2 amplitudes for both the heroin cues and neutral cues in the No-Go condition in the exercise group, particularly in the anterior cortex, were larger after exercise than those before exercise. Moreover, the N2d amplitudes were larger in the exercise group than in the control group in fronto-central scalp regions with both the heroin cues and neutral cues in the No-Go conditions during the post-test, and the N2d amplitudes were larger during the post-test than during the pre-test in the exercise group. Accordingly, N2d amplitude more clearly characterizes the beneficial effect of aerobic exercise on inhibitory control in heroin addicts than does N2 amplitude. The No-Go N2 (and N2d) amplitude has been considered a critical indicator of conflict monitoring in the early stages of inhibitory control (Falkenstein, 2006; Folstein and Van Petten, 2008). In fact, an increase in the No-Go N2 amplitude is also considered a critical indicator of successful rehabilitation (Yang et al., 2015). It is encouraging that a correlation between ΔVAS and ΔN2d amplitude was observed in the current study (*r* = 0.284). These findings indicate that acute aerobic exercise is related to reductions in cravings and improvements in inhibitory control among heroin addicts. Accordingly, improvements in inhibitory control due to aerobic exercise may be an important mechanism by which heroin cravings decrease.

A systematic review suggested that hypoactivation of some brain regions, such as the ACC, IFG, DLPFC, and inferior regions, is associated with inhibitory control abnormalities in individuals with drug addiction (including heroin addicts) (Simmonds et al., 2008). Fortunately, recent imaging studies have reported that acute aerobic exercise can enhance the activation of the DLPFC (Yanagisawa et al., 2010) and ACC (Fontes et al., 2015; Weng et al., 2017; Kim et al., 2018), suggesting that the increase in No-Go N2 (and N2d) amplitude observed in the current study can be interpreted as an external manifestation of aerobic exercise that activates these brain regions. Accordingly, we can conclude that an acute aerobic exercise intervention for heroin addicts can enhance the activation of cerebral cortexes associated with inhibitory control and functional disruption due to heroin consumption and improve the control of reckless impulses that heroin addicts tend to act upon without regard to the potentially catastrophic consequences (Baler and Volkow, 2006). This concept may be a plausible cognitive neurological mechanism by which aerobic exercise reduces heroin cravings.

### Acute Aerobic Exercise and Inhibitory Control: Spectral Power Measures

The results regarding theta 2 band spectral power revealed patterns similar to those associated with acute aerobic exercise in behavioral and N2 amplitude results. The theta 2 band spectral power outputs, particularly in the midfrontal region, were larger during the post-test than during the pre-test, in the No-Go condition with heroin cues than in the No-Go condition with neutral cues, and in the exercise group than in the control group. The exciting findings were that the Δtheta 2 spectral power was negatively correlated with ΔN2d amplitude (*r* = *−*0.314) and ΔVAS (*r* = *−*0.282) and positively correlated with ΔNo-Go accuracy (*r* = 0.440). Previous studies have suggested that increased frontal midline theta power in the No-Go condition reflects stronger inhibitory control with the Go/No-Go paradigm (Cavanagh and Frank, 2014; Harper et al., 2014; Nguyen et al., 2017), and source imaging studies have indicated that the ACC is a primary neural generator of theta-band spectral power-related No-Go N2 components (Cohen and Cavanagh, 2011; Pandey et al., 2012; Gu et al., 2019). Accordingly, the increased effects of theta 2 spectral power once again demonstrate that improvements in inhibitory control play an important role in the process by which aerobic exercise reduces heroin cravings. Unexpectedly, no significant effects of aerobic exercise on theta 2 were observed in the post-test neutral cue condition. This result may suggest that aerobic exercise is more sensitive in improving inhibitory control with heroin cues than with neutral cues. Similar trends were also exhibited in the N2d amplitude in the post-test. The findings of spectral power once again suggest that the alpha and theta bands are relevant to the Go/No-Go paradigm, and it also implies that theta 2 band spectral power is a sensitive indicator of inhibitory control in heroin addicts.

### Limitations and Future Research

To the best of our knowledge, this is the first investigation about aerobic exercise facilitating inhibitory control in heroin addicts in which behavioral and neuroelectric measures were reported. However, the results of the current study, which may be useful for future studies, should be interpreted carefully with regard to the limitations. First, the participants were recruited from the Isolated Detoxification Center and were forced to withdraw from heroin use for approximately 1 year. However, the findings from this cohort are used to guide other heroin addicts who are taking drugs, and long-term withdrawals may have certain risks due to the varying levels of brain functional impairment across participants (Rapeli et al., 2006). Future research should pay more attention to the matching of withdrawal characteristics and exercise intensity. Second, it may be risky to generalize these findings from male participants to females in heroin addict groups because sex-based differences in cravings and cognitive dysfunction have been reported (Lynch et al., 2017; Becker and Chartoff, 2019). Future research is necessary to explore the effects of aerobic exercise on cravings and inhibitory control in female heroin addicts. Third, although an attenuation in the effects of acute aerobic exercise on heroin cravings was observed 40 min after exercise, it was not possible to accurately determine the time-course effects without any attenuation of the effect of exercise. Therefore, future research should be conducted to determine the functional relationship between exercise duration and effects in heroin addicts. Last, although the effects of acute aerobic exercise may be similar to those of chronic aerobic exercise, there are differences between these types of exercise in the influences, mechanisms, and implications (Li et al., 2017; Swatridge et al., 2017), and the effects of chronic aerobic exercise on heroin cravings and inhibitory control in heroin addicts should be investigated in future research.

## Conclusion

The present study provides new evidence to support the view that acute aerobic exercise with vigorous intensity can attenuate heroin cravings and facilitate inhibitory control. Moreover, this beneficial effect of acute aerobic exercise continued for 40 min after exercise. Furthermore, the effects of aerobic exercise on inhibitory control were reflected not only in the behavioral index (i.e., No-Go accuracy) but also in the sensitivity of the electrophysiological indicators (i.e., N2d amplitude and theta 2 spectral power) relevant to early conflict monitoring. The results of this study provide a theoretical basis for future research addressing the effects of chronic aerobic exercise on the craving and cognitive function of heroin addicts.

## Data Availability Statement

All datasets presented in this study are included in the article/Supplementary Material.

## Ethics Statement

The studies involving human participants were reviewed and approved by Ethics Committee of Ningbo University. The patients/participants provided their written informed consent to participate in this study.

## Author Contributions

DW and CZ conceptualization and funding acquisition. DW and TZ methodology. TZ software. YL and JC investigation. JC data curation. DW, TZ, and Y-KC writing—original draft preparation. DW and Y-KC writing—review and editing. All authors have read and agreed to the published version of the manuscript.

## Conflict of Interest

The authors declare that the research was conducted in the absence of any commercial or financial relationships that could be construed as a potential conflict of interest.

## Footnotes

1 http://www.random.org
